# An Effective Hybrid Rescheduling Method for Wafer Chip Precision Packaging Workshops in Complex Manufacturing Environments

**DOI:** 10.3390/mi16121403

**Published:** 2025-12-12

**Authors:** Ziyue Wang, Weikang Fang, Yichen Yang

**Affiliations:** 1 School of Smart Manufacturing, Jianghan University, Wuhan 430056, China; wangziyue@jhun.edu.cn; 2 School of Mechanical Science and Engineering, Huazhong University of Science and Technology, Wuhan 430074, China; 3 Marine Design and Research Institute of China, Shanghai 200011, China; yicen1994@163.com

**Keywords:** wafer chip, precision manufacturing and packaging, rescheduling, lot streaming, dynamic events

## Abstract

With the continuous development of semiconductor manufacturing technology and information technology, the sizes of wafer chips are becoming smaller and the variety is increasing, which has put forward high requirements for wafer chip precision manufacturing and packaging workshops. On the one hand, the market demand for multiple varieties and small batches will increase the difficulty of scheduling. On the other hand, the complex manufacturing environment brings various types of dynamic events that will disrupt production plans. Accordingly, this work researches the wafer chip precision packaging workshop rescheduling problem under events of machine breakdown, emergency order inserting and original order modification. Firstly, the mathematical model for the addressed problem is established, and the rolling horizon technology is adopted to deal with multiple dynamic events. Then, a hybrid algorithm combining an improved firefly optimization framework and variable neighborhood search strategy is proposed. The population evolution mechanism is designed based on the location-updating law of fireflies in nature. The variable neighborhood search is applied for avoiding local optima and sufficiently exploring in the neighborhood. At last, the test results of comparative experiments and engineering cases indicate that the proposed method is effective and stable and is superior to the current advanced algorithms.

## 1. Introduction

Semiconductor chips, with their advantages of powerful functions and small volume, are widely used in fields such as the manufacturing industry, household appliances, medical devices, vehicles, and electrical products [1]. At present, semiconductor chips are constantly developing towards the directions of large batch and diversification [2]. Hence, higher requirements have been put forward for chip precision manufacturing, especially for the chip precision packaging production lines that are located in the middle and lower reaches of production chain [3]. Furthermore, the manufacturing system of chip precision packaging workshops is required to aim at high production efficiency, low operating costs, and timely delivery to adapt to complex and ever-changing market demands. Therefore, it poses significant challenges to the semiconductor chip precision packaging production line scheduling problem (SCPPLSP), but there are currently relatively few works on this issue.

In the semiconductor chip packaging process, wafers are processed and transported in batch forms. In order to reduce transportation costs and equipment adjustment time caused by frequent switching for different types of chips, chips of same type tend to be grouped in several batches for centralized processing, instead of being processed one by one, which is known as the lot streaming [4]. Unfortunately, under the production mode of large batches and multiple varieties, a large number of batches will drastically increase the scale of the solution space for scheduling problems [5]. Some scholars have concluded through theoretical deduction and extensive experiments that reasonable batching strategy can effectively prune the solution space for the lot streaming scheduling problem [6]. However, these existing batching strategies were specifically designed for the problems with specific characteristics and scales, which are not the primary way to solve engineering problems. An intelligent optimization algorithm is currently one of the effective ways to solve complex scheduling problems but it has low search efficiency and poor solution quality for the lot streaming scheduling problem. Therefore, it is urgent to develop reasonable and efficient intelligent optimization algorithms based on the problem characteristics to improve the quality and efficiency for the addressed SCPPLSP.

The semiconductor chip packaging process is often affected by multiple dynamic uncertainties [7]. For instance, there are many types and quantities of machines or processing units required for the chip packaging process, and hence machinery breakdown events often occur. As another example, affected by the shortage of chips in the semiconductor and electronic manufacturing market and the diversification of demands, events such as emergency orders and order cancelations often occur. The above dynamic uncertainty interferences existing in the chip packaging production line are mostly sudden events, and the rescheduling mode is commonly applied for this type of dynamic scheduling problems [8]. However, the existing studies on rescheduling problems mainly focus on the single dynamic event. Therefore, it is urgently needed to analyze the impact of dynamic event and propose rescheduling methods for the scheduling problems under multiple perturbations.

Based on the above analysis, the SCPPLSP under the multiple dynamic events will be addressed in this paper. For the scheduling optimization problems, establishing the mixed-integer linear programming (MILP) model is the foundation for analyzing the problems’ characteristics and solving combinatorial optimization problems [9]. Hence, in this work, the MILP model for the SCPPLSP is established first, and then the rescheduling method based on rolling horizon technology [10] is given to deal with the multiple different dynamic events. Firefly algorithm (FA) is designed based on simulating the flashing behaviors and movement law of fireflies in nature, and has the advantages of simple encoding, strong global search ability, flexible framework for easy improvement, and easy tuning for parameters [11]. Compared to other swarm intelligence optimization algorithms, the improved FA frameworks are more commonly used to solve complex scheduling optimization problems in practical engineering [12,13,14]. Consequently, enlightened by the design concept of standard FA, this research constructed a new improved FA framework for the SCPPLSP. To avoid local optima and strengthen global searching, the variable neighborhood descent (VND) strategy is added to the proposed improved FA framework.

The rest of this work is arranged as below. Section 2 summarizes the literatures in aforesaid fields. Section 3 characterizes the SCPPLSP and establishes the MILP model for it. In Section 4, the presented hybrid FA based on VND (HFA-VND) is explicated in detail. In Section 5, the comparison experiments and the results analysis are presented, and an engineering case verification is given. Section 6 summarizes the entire text.

## 2. Related Works

### 2.1. Semiconductor Manufacturing System

The semiconductor manufacturing system scheduling problem is an academic hotspot, and the scholars in the related fields have obtained certain study achievements [15,16]. Yang et al. [17] focused on the issues of wafer reentrance and residency time constraints. Then, they carried out the schedulability analysis on the different strategies, derived schedulability conditions, and proposed a scheduling algorithm to obtain the optimal scheduling scheme. Wang et al. [18] considered the correlation between wafer lots and production cycles in reentrant processes and put forward a fuzzy hierarchical model to adapt the production cycle of each beach. There are a variety of different performance indicators for semiconductor manufacturing systems, such as work-in-progress quantity, wafer residency time, equipment utilization rate, production cycle, operating cost, total movement volume, on-time delivery rate, energy consumption, and so on [19]. To improve the equipment utilization rate and throughput of semiconductor manufacturing systems, Siebert et al. [20] provided a batch scheduling strategy for uncertain environments. Calmels [21] addressed the multi-objective conflict problem in semiconductor manufacturing and presented a mixed-integer programming formulation along with a corresponding heuristic local search strategy. With the improvement of information technology, the analytical methods based on industrial big data technology have gradually been applied for modeling and optimization of the semiconductor manufacturing system [22,23,24,25]. Park et al. [26] addressed the large-scale production issue in semiconductor manufacturing by adopting a deep reinforcement learning approach and introducing a new state representation to adapt to changes in available resources and production requirements. Lee et al. [27] focused on the reentrant phenomenon in semiconductor manufacturing, and improved the state, action, and reward mechanisms to optimize the scheduling strategy. Although, the above studies have covered issues of reentrant processing, multi-objective optimization, uncertain disturbances, and so on, there is almost no research on SCPPLSP under the interference of multiple dynamic events.

### 2.2. Lot Streaming Scheduling

The scheduling problem with lot streaming is an integrated optimization problem of batching and scheduling, which is the NP-hard problem [28,29,30,31]. Currently, based on different batching methods, the related studies can be divided into three modes: equal-size uniform batching [32], unequal-size uniform batching [33], and variable sub-lot [34]. All of these modes have achieved certain research results. Zhang et al. [32] proposed an improved migratory bird optimization algorithm (MBO) to solve the lot streaming hybrid flowshop scheduling problem (HFSP). Wang et al. [35] focused on the integrated problem of batch processing and lot streaming in a two-stage HFSP and adopted a heuristic algorithm to minimize the total weighted completion time. Nejati et al. [36] studied the HFSP with lot streaming under multi-stage unequal-size lots with shift constraints. Considering the scenario of interleavable sublots, Nejati et al. [37] studied a two-stage assembly line with lot streaming and unequal-size lots. They took the makespan as the optimization objective, and applied the genetic algorithm (GA) and simulated annealing (SA) algorithm, respectively. For a special form of hybrid flow shop scheduling problem with lot streaming and multi-stage unequal-size lots, Lalitha et al. [38] established the MILP model and proposed a related heuristic rule for solving. For the lot streaming scheduling problem, Novas [39] designed a novel constraint method and determined the number of sublots for all lots in the scheduling scheme. A large number of batch divisions will greatly enlarge the size of solution space [6,40], and reasonable batching strategy can effectively improve the solving efficiency. Vivek et al. [41] used a heuristic method to evaluate 11 batching strategies and obtain the optimal batching method. However, the batching strategy is usually specifically designed for the problems with specific characteristics and scales. Hence, proposing an efficient optimization algorithm is an effective way to solving scheduling optimization problem with lot streaming.

### 2.3. Dynamic Scheduling

The complex production environment and diverse market demands [42] will disrupt the scheduling plans formulated by the manufacturing system. The scholars dedicated to dynamic scheduling problems mainly focus on scheduling methods, such as rescheduling [43], inverse scheduling [44], and robust scheduling [45,46,47]. Rescheduling mode is often adopted for the unexpected dynamic events that have significant impact on production plans. Zhang et al. [10] presented a rescheduling method based on a multi-objective MBO for HFSP. He et al. [48] provided a rescheduling model for hybrid flow shop to address the problem of urgent order insertion and used the NSGA-III algorithm to solve it. Inverse scheduling mode involves adjusting controllable resources and processing parameters to meet production requirements instead of changing the production plan, which is suitable for the unexpected dynamic events that have small-scale impact on production plans [49]. Mou et al. [50] adopted an inverse scheduling mode and studied a hybrid algorithm for the energy-efficient scheduling problem. Zhang et al. [51] proposed an efficient hybrid integer and a categorical particle swarm optimization algorithm for the multi-mode multi-project inverse scheduling problem in the turbine assembly workshop. For random uncertain disturbances, robust scheduling strategies are often employed. Qiao et al. [52] proposed a robust real-time scheduling strategy that combined a real-time controller with offline scheduling. To tackle the problem of uncertain processing times in semiconductor manufacturing systems, Liu et al. [53] proposed a three-stage multi-objective robust optimization method to enhance system stability. Preventive maintenance strategy is one of the robust scheduling methods, which is used to handle dynamically predictable events. Lee et al. [54] designed a preventive maintenance optimization model to prevent the impact of downtime events on semiconductor manufacturing systems. Obviously, there have been certain studies on the dynamic scheduling problems under a single dynamic event, but the related research on multiple types of uncertain events is paid little attention, especially for the scheduling problem in semiconductor chip packaging production lines.

## 3. Problem Formulation

### 3.1. Problem Description

SCPPLSP can be equivalent to an HFSP with the lot streaming processing mode and the interference of multiple dynamic events. In this research, the most common dynamic events in chip packaging production lines, namely machine breakdown, emergency order inserting, and original order modification, are considered in SCPPLSP. Then, the SCPPLSP under multiple dynamic events can be described as follows: the chips of several types in the order need to be continuously processed in several processing stages, and the processing steps for all the chips are the same. In each processing stage, there are several same processing units or machines available for selection. The chips of each type are divided into sub-lots with the same number. Each sub-lot that has been completed at a certain processing stage can be immediately carried to the next process stage, while the different sub-lots can be processed simultaneously at different stages, which accelerates the operation of the production line and improves production efficiency. In SCPPLSP, the sub-lots are treated as the smallest processing units for scheduling. SCPPLSP aims to minimize the makespan as the optimization objective. Here, the following hypothesis settings are given:

- To avoid the frequent switching, all sub-lots of each type of chip can only be processed continuously by the same processing unit or machine at each stage;
- All the processing unit can only deal with one sub-lot simultaneously, and each sub-lot can only be processed by one processing unit;
- There is no waiting time limit for each sub-lot between the adjacent processing stages;
- The capacity of the buffer between the adjacent processing stages is usually set to be much larger than the number of chips in process, so its impact on production plan and rhythm can be approximately ignored;
- The setup times can be approximated to be sequence independent and have been calculated within the processing times;
- The transportation time between adjacent processing stages has been calculated in the processing time;
- Machine breakdown events randomly occur at any time, and the maintenance time can be accurately predicted;
- Emergency order inserting events can randomly occur randomly at any time, and a certain number of sub-lots will be added to the production plan;
- Original order modifications can randomly occur randomly at any time, and a certain number of sub-lots will be removed from the production plan.

### 3.2. Rescheduling Mode

The dynamic events described in this work, namely machine breakdown, emergency order inserting, and original order modification, can be treated as sudden events, and it is generally difficult to accurately predict the exact time when these dynamic events will occur. Therefore, the rescheduling mode is adopted here. The rescheduling mode can be described as follows: when a dynamic event occurs, the unprocessed operations are rescheduled for the optimization of performance indicators. The rolling horizon method is commonly used for the rescheduling problem, which can divide the timeline into several parts [13]. In this research, different types of dynamic events occur at different times, so at each time when a dynamic event occurs, namely the rescheduling time, all the operations of each sub-lot need to be analyzed and judged based on the rolling horizon theory.

After a dynamic event occurs, all operations of each sub-lot will be divided into different state sets based on their processing status. State set 1 includes the processed operations and the ones that have been started on the non-faulty processing units. State set 2 includes the unprocessed operations. The operation being processed on the faulty processing unit or machines belongs to State set 3, which can be processed continuously after the faulty processing unit is restored. For the urgently inserted order, all the operations of the chips in the order should be added to the State set 2, which will be treated as a new chip type in rescheduling plan. For the modified original order, all the operations of the canceled chips will be canceled from the rescheduling plan. Hence, only the operations in State set 2 should be rearranged at the rescheduling time.

**Figure 1:**
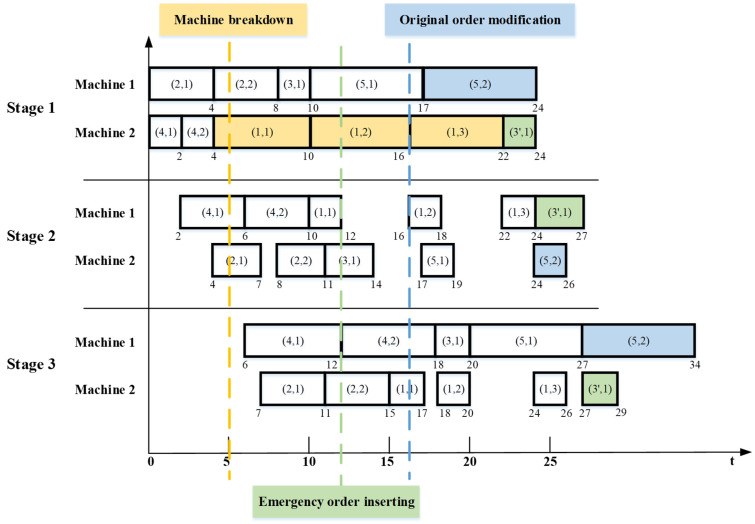
The rescheduling mode.

Here, a simple instance is given to show the rescheduling mode under three dynamic events described above, as shown in Figure 1. (*j*,*e*) represents the sub-lot *e* of the chip type *j*. At rescheduling time 1, machine 2 at the first processing stage breaks down. The operations (1,1), (1,2), (1,3) on the faulty machine, which are in the yellow box, belong to State set 3, while the operations (2,1), (4,1), (4,2) at stage 1 and the operations (2,1), (2,2), (4,1), (4,2) at stage 2 belong to State set 1. The other operations belong to State set 2. At rescheduling time 2, the emergency order is inserted and one sub-lot of chip type 3 is added, namely operation (3′,1), which is in the green box and is treated as a new type for rescheduling. At rescheduling time 3, the original order is modified and the operation (5,2) is removed, as shown in the blue box. At this time, the operation (3′,1) at stage 1, the operations (3′,1), (5,1) at stage 2, and the operations of (3,1), (3′,1), (5,1) at stage 3 belong to State set 2, which should be rescheduled.

### 3.3. Mathematical Model

According to the problem description of SCPPLSP and the rescheduling mode based on the rolling horizon method, the MILP model for SCPPLSP is established. Firstly, the related symbols are defined as in Abbreviations.

Then, give the MILP model for SCPPLSP:

- **Objective:**

(1) $$f=tC_{max}=\max\left(tC_{1},tC_{2},tC_{3},\ldots ,tC_{n}\right)$$

- **Subject to:**

(2) $$\sum_{j=1}^{m_{k}}b{D^{\prime }}_{i,j,k}=1\;\forall i\in J,\;k\in M,\;j\in M_{k}$$

(3) $$t{C^{\prime }}_{i,e,k}-t{S^{\prime }}_{i,e,k}=tP_{i,k}\;\;\;\forall O_{i,e,k}\in State\;2$$

(4) $$t{S^{\prime }}_{i,e,k+1}-t{C^{\prime }}_{i,e,k}\geq 0\;\forall O_{i,e,k}\in State\;2$$

(5) $$t{S^{\prime }}_{i,e+1,k}-t{C^{\prime }}_{i,e,k}\geq 0\;\forall O_{i,e,k}\in State\;2$$

(6) $$b{S^{\prime }}_{i\prime ,i,k}+b{S^{\prime }}_{i,i\prime ,k}\leq 1\;\forall O_{i,e,k},O_{i\prime ,e\prime ,k}\in State\;2$$

(7) $$b{S^{\prime }}_{i,i\prime ,k}+b{S^{\prime }}_{i\prime ,i,k}\leq b{D^{\prime }}_{i,j,k}+b{D^{\prime }}_{i\prime ,j,k}\;\forall O_{i,e,k},O_{i\prime ,e\prime ,k}\in State\;2$$

(8) $$b{D^{\prime }}_{i,j,k}+b{D^{\prime }}_{i\prime ,j,k}-1\leq b{S^{\prime }}_{i,i\prime ,k}+b{S^{\prime }}_{i\prime ,i,k}\;\forall O_{i,e,k},O_{i\prime ,e\prime ,k}\in State\;2$$

(9) $$t{S^{\prime }}_{i,1,k}-t{C^{\prime }}_{i\prime ,l_{i\prime },k}+Q\times (3-b{S^{\prime }}_{i,i\prime ,k}-b{D^{\prime }}_{i,j,k}-b{D^{\prime }}_{i\prime ,j,k})\geq 0\;\forall O_{i,e,k},O_{i\prime ,e\prime ,k}\in State\;2$$

(10) $$t{S^{\prime }}_{i,e,k}=tS_{i,e,k}\;t{C^{\prime }}_{i,e,k}=tC_{i,e,k}\;\forall O_{i,e,k}\in State\;1$$

(11) $$b{D^{\prime }}_{i,j,k}=bD_{i,j,k}\;b{S^{\prime }}_{i,i\prime ,k}=bS_{i,i\prime ,k}\;\forall O_{i,e,k},O_{i\prime ,e\prime ,k}\in State\;1\cup State\;3$$

(12) $$t{S^{\prime }}_{i,e,k}=tS_{i,e,k}\;t{C^{\prime }}_{i,e,k}=tC_{i,e,k}+tB-tR\;\forall O_{i,e,k}\in State\;3$$

(13) $$b{D^{\prime }}_{i,j,k}\in \{0,1\}\;b{S^{\prime }}_{i,i\prime ,k}\in \{0,1\}\;\forall O_{i,e,k},O_{i\prime ,e\prime ,k}\in State\;2$$

Equation (1) states that the optimization objective of the MILP model for SCPPLSP is the makespan. Constraints (2)–(9) provide the constrained condition for SCPPLSP in rescheduling. Constraints (10) and (11) state that the rescheduling plans of the operations in State set 1 are the same as the original plans of them. Constraint (12) states that, in the rescheduling plan, the operation belonging to State set 3 will be processed on the faulty machine continuously after the faulty machine is restored. Constraint (13) provides the value ranges for the two decision variables in rescheduling, respectively.

## 4. Hybrid Firefly Algorithm Based on Variable Neighborhood Descent

### 4.1. Algorithm Framework

As described in Section 1, FA has the advantages of simple encoding, strong global search ability, flexible framework for easy improvement, and easy tuning for parameters, which is more commonly improved to solve actual complex scheduling problems in production shop. Accordingly, an improved FA framework is proposed here to solve SCPPLSP. The design concept of standard FA originates from the movement patterns of firefly in nature, where the darker individuals will move closer to the brighter ones. Inspired by the FA concept, in our improved framework, the suboptimal individuals move toward more optimal ones by designing movement operator, while the random perturbation is introduced to enhance the population diversity. To avoid local optima and strengthen global searching, the local search operation based on VND is adopted in above algorithm framework. The algorithm flow of HFA-VND is shown in Algorithm 1:
**Algorithm 1** The algorithm flow of HFA-VNDSet the population size *ps* and the maximum number of iterations for VND *maxtsIter*;**Population initialization**: Generate initial population containing *ps* individuals;**Individuals movement**: In stage β, compare all the individuals pairwise and make suboptimal individuals move toward more optimal ones, while in stage α, the random perturbations are used to avoid premature entry into local optima;**Local search**: Select several optimal individuals to conduct the local search operation to ensure sufficient mining in their neighborhood;Go to step 6 if the termination condition is met; go to step 3 otherwise;Output the results.

### 4.2. Individual Description

As described in Section 3.1, in the SCPPLSP, all sub-lots can only be processed by one processing unit, and all the chips have the same processing sequence. Therefore, we adopt an encoding mechanism based on the sequence of chip types, which represents the processing order of chip types in the initial stage and has been effectively used for solving HFSP. The encoding sequence can be represented as: {*E*(1), *E*(2), …, *E*(*n*)}, where *E*(*i*) is the index of the chip type.

According to the problem characteristics of SCPPLSP and the above encoding structure, it is required that the decoding mechanism should solve the following two sub-problems: one is the sequence of chip types at other processing stages, and the other is the machine selection for different chip types at each stage. In order to shorten the waiting times of chips and the idle times of machines, two heuristic rules are adopted as below:

- The “first come, first processed for sub-lot” rule: a certain sub-lot of a certain chip type that has been processed at a certain stage does not need to wait for the other subsequent sub-lots of this chip type, and can directly start to be processed at the subsequent stage;
- The “first idle machine, first processing” rule: the sub-lot that needs to be processed should be assigned on the first idle machine at this stage at this time.

Based on the above analysis, the decoding mechanism can be described in Algorithm 2:
**Algorithm 2** The algorithm flow of the decoding mechanism1.All the operations of all the sub-lots should be divided into three state sets based on the rolling horizon technology;2.Find the earliest processing stage s’ (1 ≤ s’ ≤ m) among all the operations in State set 2;3.Record the number *n_k_* (*k*∈{*s’*, *s’* + 1…, *m*}) of the chip types belonging to State set 2 at each stage;4.Sort the chip types belonging to State set 2 at the stage *s’* based on the encoding sequence;5.**For** *i* **in** {1,2,…, *n_s’_*} **do**
6.        Select the first idle machine, and process all the sub-lots of the chip type *i* sequentially on this machine;7.        Calculate the processing times of these sub-lots, and use the completion time of the last sub-lot to update the machine idle time;8.**End**9.**For** *k* **in** {*s’* + 1…, *m* } **do**10.      Sort the chip types belonging to State set 2 at the stage *k* according to the ascending order of completion time of the first sub-lot of each type;11.      **For** *i* **in** {1,2,…, *n_k_*} **do**12.              Select the first idle machine, and process all the sub-lots of chip type *i* sequentially on this machine;13.              Calculate the processing times of these sub-lots, and use the completion time of the last sub-lot to update the machine idle time;14.      **End**15.**End**

### 4.3. Population Initialization

To ensure the diversity and superiority of initial individuals, a hybrid initialization strategy based on the integrating random rule and NEH rule is proposed here to generate the initial population. To avoid the long waiting time, two heuristic rules are adopted, respectively, in the NEH rule, namely the longest processing time rule (LPT) and the longest processing time at the first stage rule (LPTF). The hybrid initialization strategy can be described in Algorithm 3:
**Algorithm 3** The algorithm flow of the hybrid initialization strategyRandomly generate *ps*-2 individuals;Generate an individual according to the NEH rule based on LPT:Calculate the total processing times of all chip types, and sort the chip types based on their descending sequence;Successively take out the chip types from the above sequence and insert them into all available positions in order to obtain the optimal subsequence;Proceed until the last chip type is inserted into a suitable position, and the optimal individuals are obtained;Generate an individual according to the NEH rule based on LPTF:Calculate the processing times of all chip types at the first stage, and sort the chip types based on their descending sequence;Successively take out the chip types from the above sequence and insert them into all available positions in order to obtain the optimal subsequence;Proceed until the last chip type is inserted into a suitable position, and the optimal individuals are obtained;The above ps individuals are used to form the initial population.

### 4.4. Individual Movement

**Figure 2:**
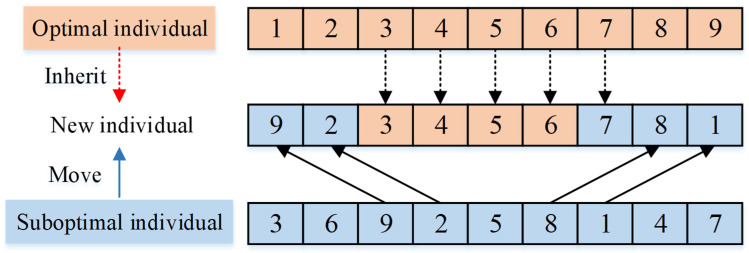
The individual movement strategy based on PBX.

**Figure 3:**
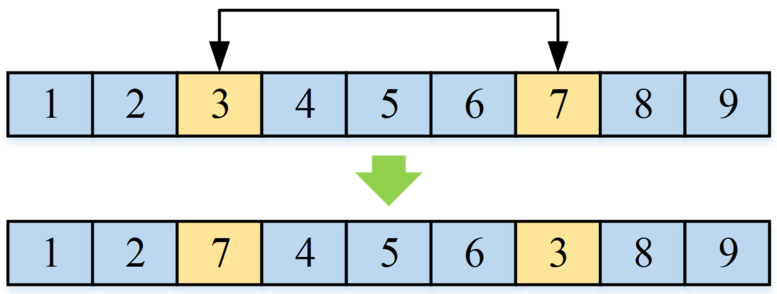
The random perturbation operators: swap operator.

**Figure 4:**
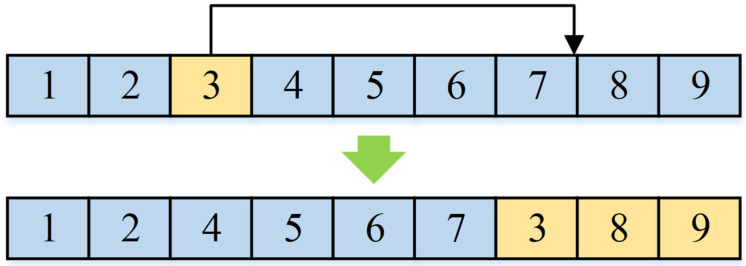
The random perturbation operators: insertion operator.

According to the design thought of standard FA, the individual movement process includes two stages: in the stage β, the suboptimal individuals move closer to more optimal ones, and in the stage α, the individual is randomly modified to avoid local optima. As described in Section 4.1, the improved FA framework proposed in the paper is designed inspired by the core idea of the standard FA. As an improvement, we do not use the firefly position update equation of standard FA to achieve individual movement. Instead, according to the characteristics of the addressed SCPPLSP and the encoding structure described in Section 4.2, in the stage β of the improved algorithm framework, a position-based crossover (PBX) operator is used to realize the individual movement. As shown in Figure 2, by using the PBX operator for two individuals, the new individual will inherit the excellent genetic fragments from the better individual, which achieves the movement of the suboptimal individual towards the better one. Then, in stage α of the improved algorithm framework, the random perturbation based on swap operator and insertion operator is applied for avoiding local optima, as shown in Figure 3 and Figure 4. In this research, only one operator is selected for random variation for each individual, and here the probability of both above operators being selected is 50%.

### 4.5. Local Search

To avoid local optima and sufficiently explore the neighborhood of the optimal individuals, the VND strategy is adopted and introduced to the improved FA framework. The design concept of VND can be described as follows: if the more optimal individual cannot be mined from the current neighborhood after a certain number of iterations, the next neighborhood will be selected for local search, while if the more optimal individual is mined, the search operation will turn back to the first neighborhood. The neighborhood structures selected for VND in this work are the swap operator and insertion operator described in Section 4.4. The pseudocode of the VND is shown in Algorithm 4:
**Algorithm 4** The algorithm flow of VND*r* = 1, *iter* = 0;**While** (*isStop* == *False*)        *X’* = *N_r_*(*X*);        **If** *f*(*X’*) < *f*(*X*)                *X’_i_* = *X_i_*, *r* = 1, *iter* = 0;        **Else**                *iter*++;        **End**        **If** *iter* > *maxtsIter*                **If** *r* < 2                        *r*++, *iter* = 0;                **Else**                        *r* = 1, *iter* = 0, *isStop* = *true*;                **End**        **End****End**

## 5. Experimental Study

### 5.1. Experimental Design

There has been no benchmark for the addressed SCPPLSP at present, so we selected the test instances randomly generated to evaluate the algorithm performance, which is widely applied for the complex scheduling problem. To simulate the actual processing environment in the semiconductor chip packaging production line, 5 different scales of numbers of the chip types and 2 different scales of numbers of the processing stages are selected to form the test instances of 10 scales. In total, 10 specific test instances are randomly generated for each scale, so a total of 100 test instances are obtained. The occurrence times of these three dynamic events are also randomly valued in the integer interval [1, 99], and the duration of machine breakdown is randomly chosen in the integer interval [20, 99]. The other parameters of these instances are listed in Table 1.

All the algorithms for comparison tests are realized by Visual C++ language in Visual Studio 2015 on a computer with AMD Ryzen 7 5800H CPU at 3.20 GHz and 16G RAM. The test study in this section will use the best *C_max_* and the relative percentage increase (*RPI*) obtained from different algorithms as the references to verify the superiority of HFA-VND. The *RPI* for the algorithm ***X*** is formulated as:

(14) $$RPI(X)=(tC_{X-best}-tC_{best})/tC_{best}\times 100\%$$

where *C_X__-best_* and *C_best_* are the best solutions, respectively, obtained from the algorithm ***X*** and all the algorithms. In order to respond promptly to dynamic events with minimal impact on production efficiency, the algorithm execution time limit is set as the algorithm termination condition. Here, the maximum execution time is designed as 10 × *m* × *n* (ms), which can ensure that the production plans will be adjusted within tens of seconds after the dynamic events occur and meet the production demands for SCPPLSP in actual working conditions.

### 5.2. Algorithm Comparison

To test the performance of HFA-VND, this section introduces the existing advanced algorithms for comparative testing. The introduced algorithms are GA [37], genetic algorithm and tabu search (GATS) [55], genetic algorithm and variable neighborhood search (GAVNS) [56] and MBO [32], which are widely applied for the complex scheduling problem and achieve excellent results.

**Figure 5:**
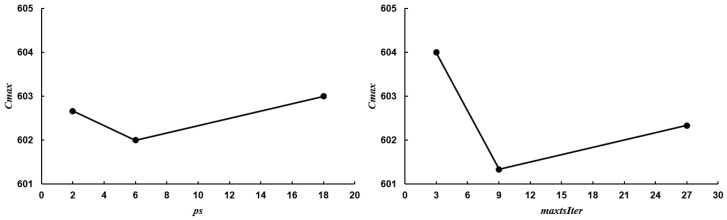
Taguchi test results for HFA-VND.

The population size *ps* and the maximum number of iterations for VND *maxtsIter* are selected through Taguchi experimental testing, which is specially used for the parameter selection and calibration in the field of scheduling optimization. The test results are shown in Figure 5. Seen from Figure 5, population size *ps* and the maximum number of iterations *maxtsIter* are set as 6 and 9, respectively. In GA, GATS and GAVNS, the algorithm termination times are set as the same as HFA-VND. The other parameters of them are also chosen after Taguchi experimental testing. The parameters in MBO are set according to the references [32].

**Figure 6:**
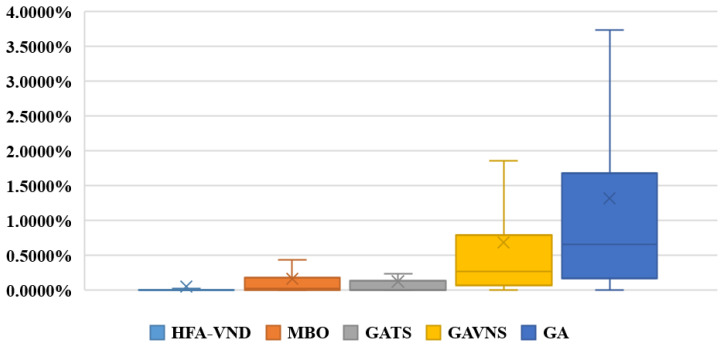
The boxplot for the averages of *RPI* obtained from different algorithms under each instance.

The 100 test instances generated according to Section 5.1 are used for comparative testing. Under each instance, every algorithm need to be tested 10 times. Then, the best *C_max_* and the averages of *RPI* obtained from each algorithm under each test instance are recorded in Table 2. The statistical information (the numbers of best *C_max_* or *RPI*, the averages of *RPI* and the variance of *RPI*) of test results for each algorithm is recorded at the bottom of Table 2. The boxplot for the averages of *RPI* obtained from different algorithms under each instance is shown in Figure 6.

In the Table 2, the bolded parts represent the best results obtained from these different algorithms under the corresponding instances. The comparison test results and the statistical information for different algorithms in Table 2 show that HFA-VND can achieve 97 best *C_max_* and 98 best averages of *RPI* from the 100 instances. On the contrary, MBO, GATS, GAVNS, and GA cannot achieve the best results under dozens of instances. Moreover, the averages of *RPI* for each instance obtained from the other algorithms are inferior to those solved from HFA-VND. Through the further analysis for the test results in Table 2, GATS and MBO, which are often designed for complex scheduling problems, are superior to the common optimization algorithms (GAVNS and GA), and the hybrid algorithms (MBO, GATS, and GAVNS) are superior to the single algorithm (GA). Seen from the boxplot shown in Figure 6, the test results obtained from HFA-VND have more concentrated data distribution and fewer outliers. Therefore, it can be proved that HFA-VND proposed in this work is effective and stable for solving SCPPLSP under the multiple dynamic events and is superior to the current advanced optimization algorithms in the field of workshop scheduling.

### 5.3. Case Study

**Figure 7:**
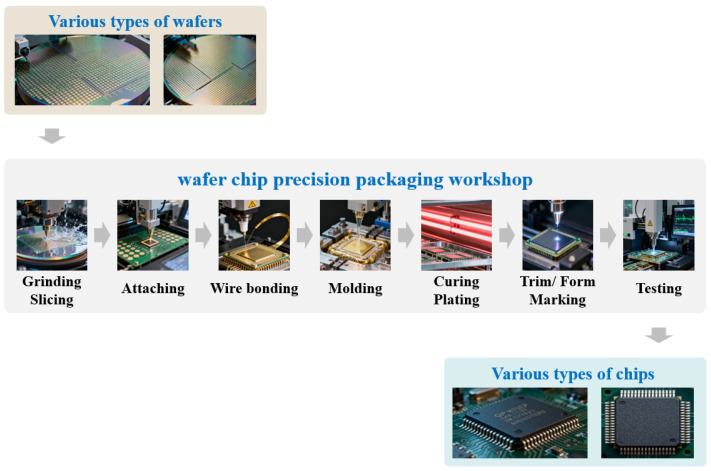
The semiconductor chip packaging processing flow.

In this section, the actual processing parameters and order data from a typical semiconductor chip packaging enterprise are used for a case test. In this enterprise, the semiconductor chip packaging production line contains 7 main processing stages, namely the grinding and slicing, attaching, wire bonding, molding, curing and plating, Trim/Form and marking, and testing, as shown in Figure 7. In the packaging production line, various types of wafers are packaged to various types of semiconductor chips. In the production workshop of the enterprise, each processing stage contains several packaging equipment or processing units.

Here, we use an actual order of this enterprise, which includes 60 types of chips, for a case test. In the test, the three types of dynamic events are simulated to occur randomly during the processing. To verify the effectiveness and superiority of the proposed method, MBO, GATS, GAVNS, and GA are introduced again for comparative testing. Moreover, the scheduling rules LPT and LPTF, which are currently being used by the enterprise, are also added to the comparative testing. The algorithm settings are the same as those in Section 5.2. The comparison test results are recorded in Table 3, and the bolded part represents the best result obtained from these different algorithms under the case test. The test results of actual case in Table 3 show that the HFA-VND can achieve the best scheduling result. As the same as Section 5.2, MBO is also superior to the other algorithm for actual case, and hybrid algorithms also perform better than the single algorithm. Comparing the scheduling rules LPT and LPTF, intelligent optimization algorithm can obtain the more optimal results. It can be seen that the makespan obtained from the HFA-VND can decrease by about 7% compared to LPT and LPTF. The case tests show the HFA-VND is effective for the SCPPLSP in the actual environment and can improve the production efficiency for semiconductor chips’ packaging enterprise.

## 6. Conclusions

This paper proposed an HFA-VND to solve the SCPPLSP under multiple dynamic events. Firstly, the SCPPLSP is described in detail. After analysis on the three types of dynamic events, namely machine breakdown, emergency order inserting, and original order modification, the rescheduling mode based on rolling horizon technology is adopted. Based on the problem characteristics, the MILP model for SCPPLSP is established. Then, inspired by the design idea of standard FA, an improved FA framework is constructed. In order to efficiently describe the individuals, an encoding mechanism based on the sequence of chip types is used. Correspondingly, to save on the waiting times of sub-lots and the idle times of machines, the decoding mechanism based on two heuristic rules is presented to obtain the rescheduling plan. To ensure the diversity and superiority of the initial individuals, the random individuals and the NEH rule based on LPT and LPTF are applied to generating the initial population. In the process of population evolution, the individual movement strategy based on PBX improves the solutions quality, and the random perturbation based on the swap operator and insertion operator is introduced to enhance search capability. The local search mechanism based on VND for the optimal individuals ensures the sufficient exploration in their neighborhood. At last, the comparative test results under the 100 instances generated randomly show that the HFA-VND is effective for SCPPLSP and is superior to the current advanced algorithms, namely MBO, GATS, GAVNS, and GA. Additionally, a case test from semiconductor chips’ precision packaging enterprise verified that the HFA-VND is effective for the actual production, and can accelerate the production efficiency for the enterprise.

In future studies, more types of dynamic events will be introduced, such as fuzzy processing time, lack of raw materials, periodic preventive maintenance, and so on. In addition, more optimization objectives will be involved, like energy consumption, utilization rate of equipment, production cost and tardiness. Additionally, the further improvement of the optimization algorithms will still be one of the important research directions.

## Figures and Tables

**Table 1:** The instances parameters.

Parameters	Values
*n*	{20, 40, 60, 80, 100}
*s*	{5, 10}
*m_k_*	integer in [5, 10]
*tP_i,k_*	integer in [10, 20]
*l_i_*	integer in [5, 10]

**Table 2:** The comparison experimental results.

Instance *m* × *n*	HFA-VND		MBO		GATS		GAVNS		GA	
	*C_max_*	*RPI* (%)	*C_max_*	*RPI* (%)	*C_max_*	*RPI* (%)	*C_max_*	*RPI* (%)	*C_max_*	*RPI* (%)
20 × 5	**480**	**0.1875**	**480**	0.8125	**480**	0.4583	484	1.4583	486	1.9792
	**517**	**0.0000**	**517**	0.3482	**517**	0.0193	518	1.1605	521	2.0309
	**477**	**0.0000**	**477**	0.0629	**477**	**0.0000**	**477**	0.0839	**477**	0.3145
	**321**	**0.0000**	**321**	0.7788	**321**	**0.0000**	322	2.2741	329	4.1745
	**476**	**0.0000**	**476**	0.2311	**476**	**0.0000**	**476**	0.5252	481	2.0588
	**710**	**0.0000**	**710**	0.0845	**710**	**0.0000**	**710**	0.0423	**710**	0.0423
	**854**	**0.0000**	**854**	**0.0000**	**854**	**0.0000**	**854**	0.0234	**854**	0.0585
	**543**	**0.0000**	**543**	**0.0000**	**543**	**0.0000**	**543**	0.0368	**543**	0.1105
	**372**	**0.0000**	**372**	0.6989	**372**	**0.0000**	**372**	0.4839	375	1.3172
	**936**	**0.0000**	**936**	**0.0000**	**936**	**0.0000**	**936**	0.0107	**936**	0.0214
20 × 10	**1029**	**0.0000**	**1029**	**0.0000**	**1029**	**0.0000**	**1029**	**0.0000**	**1029**	**0.0000**
	**848**	**0.0000**	**848**	0.1533	**848**	**0.0000**	**848**	0.0825	**848**	0.0708
	**772**	**0.0000**	**772**	0.1943	**772**	**0.0000**	**772**	0.2073	**772**	0.5829
	**1104**	**0.0000**	**1104**	**0.0000**	**1104**	**0.0000**	**1104**	**0.0000**	**1104**	0.5072
	**966**	**0.4037**	973	0.9627	968	0.5901	972	1.9048	974	3.4990
	**519**	**0.0000**	**519**	**0.0000**	**519**	**0.0000**	**519**	0.3083	**519**	1.0405
	**1101**	**0.0000**	**1101**	0.1817	**1101**	0.0091	**1101**	0.8629	**1101**	2.6431
	**927**	**0.0000**	**927**	0.0647	**927**	**0.0000**	**927**	0.1834	**927**	0.7443
	**984**	**0.0000**	**984**	0.0610	**984**	**0.0000**	**984**	0.0813	**984**	0.2642
	**662**	**0.0000**	**662**	0.6495	**662**	0.1511	668	2.5680	693	5.7100
40 × 5	**503**	**1.1730**	506	1.7495	508	1.7296	517	5.6461	539	8.8469
	930	**0.1184**	930	0.2476	**929**	0.1830	940	2.9064	963	5.6189
	**1379**	**0.0000**	**1379**	**0.0000**	**1379**	**0.0000**	**1379**	**0.0000**	**1379**	0.1015
	**1667**	**0.0000**	**1667**	**0.0000**	**1667**	**0.0000**	**1667**	**0.0000**	**1667**	**0.0000**
	**944**	**0.0106**	**944**	0.9216	**944**	0.2331	948	1.2606	954	2.3093
	**609**	**0.0000**	**609**	**0.0000**	**609**	**0.0000**	**609**	0.0821	**609**	0.3777
	**601**	**0.0000**	602	0.4825	**601**	0.2163	604	0.8319	608	1.5973
	**771**	**0.1427**	**771**	0.4410	772	0.3891	776	1.8029	786	3.7354
	**694**	**0.1009**	695	0.6484	**694**	0.1873	702	2.1470	706	3.1844
	**800**	**0.0000**	**800**	**0.0000**	**800**	**0.0000**	**800**	0.2250	801	0.9250
40 × 10	**1401**	**0.0000**	**1401**	**0.0000**	**1401**	**0.0000**	**1401**	0.5639	**1401**	1.7987
	**1541**	**0.0000**	**1541**	0.1363	**1541**	0.0454	**1541**	0.2141	1544	0.3504
	**1473**	**0.0000**	**1473**	0.0815	**1473**	0.0950	**1473**	0.3530	1478	0.5635
	**1788**	**0.0000**	**1788**	0.0224	**1788**	**0.0000**	**1788**	0.5817	1794	2.0246
	**1442**	**0.0000**	**1442**	0.0624	**1442**	**0.0000**	**1442**	0.1526	1447	0.9015
	**1645**	**0.0243**	**1645**	0.2736	**1645**	0.1216	1647	0.3404	1650	0.5410
	**1180**	**0.1271**	1181	0.1695	1182	0.1695	1190	2.3220	1211	4.5763
	**1439**	**0.0000**	**1439**	**0.0000**	**1439**	**0.0000**	**1439**	0.2224	**1439**	0.7088
	**1666**	**0.1200**	**1666**	0.4742	**1666**	0.2221	1681	2.3229	1732	5.0360
	**1365**	**0.0000**	**1365**	0.1465	**1365**	0.0293	**1365**	0.1612	**1365**	0.4762
60 × 5	**2449**	**0.0000**	**2449**	0.0817	**2449**	**0.0000**	**2449**	**0.0000**	**2449**	0.0204
	**987**	**0.0101**	988	0.2128	**987**	0.1621	**987**	0.6586	996	1.7021
	**2198**	**0.0000**	**2198**	**0.0000**	**2198**	0.0045	**2198**	0.1365	**2198**	0.1683
	**1805**	**0.0000**	**1805**	**0.0000**	**1805**	**0.0000**	**1805**	**0.0000**	**1805**	**0.0000**
	**2266**	**0.0000**	**2266**	**0.0000**	**2266**	**0.0000**	**2266**	0.1721	**2266**	0.1765
	**2056**	**0.0000**	**2056**	**0.0000**	**2056**	**0.0000**	**2056**	0.0049	**2056**	0.0632
	**1293**	**0.0000**	**1293**	0.0232	**1293**	**0.0000**	**1293**	0.2320	**1293**	0.1469
	**1933**	**0.0000**	**1933**	**0.0000**	**1933**	0.0052	**1933**	0.3001	**1933**	0.4190
	**2064**	**0.0000**	**2064**	**0.0000**	**2064**	**0.0000**	**2064**	0.0581	**2064**	0.4457
	**2093**	**0.0000**	**2093**	0.0096	**2093**	**0.0000**	**2093**	0.0334	**2093**	0.0956
60 × 10	**2548**	**0.0078**	**2548**	0.1138	2550	0.2198	2556	0.9027	2569	1.4717
	**2312**	**0.0087**	**2312**	0.0173	**2312**	0.0216	**2312**	0.1125	2314	0.1557
	**2493**	**0.0000**	**2493**	**0.0000**	**2493**	**0.0000**	**2493**	**0.0000**	**2493**	**0.0000**
	1318	1.0687	**1310**	**0.8779**	1323	1.7634	1354	4.6947	1390	7.7481
	**2294**	**0.0000**	**2294**	0.1351	**2294**	0.2180	2312	2.5327	2372	4.5205
	**2303**	**0.0043**	**2303**	0.0261	**2303**	0.0261	**2303**	0.8033	**2303**	1.3200
	**2275**	0.0132	**2275**	**0.0000**	**2275**	0.2022	2286	1.0022	2296	1.4374
	**2807**	**0.0000**	**2807**	0.0143	**2807**	**0.0000**	**2807**	0.1603	**2807**	0.1176
	**2091**	**0.0000**	**2091**	**0.0000**	**2091**	**0.0000**	**2091**	0.0191	**2091**	0.0670
	**2314**	**0.0000**	**2314**	**0.0000**	**2314**	**0.0000**	**2314**	0.4322	2316	0.6698
80 × 5	**3032**	**0.0000**	**3032**	**0.0000**	**3032**	**0.0000**	**3032**	0.1682	**3032**	0.1748
	**2743**	**0.0000**	**2743**	0.0292	**2743**	0.0146	**2743**	0.1568	2746	0.1969
	**2963**	**0.0000**	**2963**	**0.0000**	**2963**	**0.0000**	**2963**	0.0540	**2963**	0.2565
	**1028**	**0.3307**	1030	0.5545	1031	0.6712	1038	1.8580	1045	2.9961
	**1466**	**0.0205**	**1466**	0.1296	**1466**	0.1501	1467	0.7503	1472	0.9891
	**2953**	**0.0000**	**2953**	**0.0000**	**2953**	**0.0000**	**2953**	**0.0000**	**2953**	0.0847
	**3049**	**0.0000**	**3049**	**0.0000**	**3049**	**0.0000**	**3049**	0.0656	**3049**	0.0918
	**1029**	**0.1069**	1031	0.4859	**1029**	0.5831	1035	1.4383	1036	2.3032
	**2933**	**0.0000**	**2933**	**0.0000**	**2933**	**0.0000**	**2933**	0.0034	**2933**	0.0136
	**3411**	**0.0000**	**3411**	**0.0000**	**3411**	**0.0000**	**3411**	0.0967	**3411**	0.0850
80 × 10	**3330**	**0.0210**	**3330**	0.0631	**3330**	0.0300	3336	0.4174	3340	0.7838
	**3001**	**0.0000**	**3001**	0.0233	**3001**	0.0133	3009	0.6698	3031	1.6028
	**3093**	**0.0000**	**3093**	0.4753	**3093**	**0.0000**	**3093**	0.1843	3094	0.6790
	**3254**	**0.0000**	**3254**	**0.0000**	**3254**	0.0277	**3254**	0.3534	3263	0.6669
	**3335**	**0.0000**	**3335**	0.0090	**3335**	0.0300	**3335**	0.5607	3339	0.8126
	**3390**	**0.0000**	**3390**	**0.0000**	**3390**	**0.0000**	**3390**	0.1917	**3390**	0.5575
	**3220**	**0.0000**	**3220**	**0.0000**	**3220**	**0.0000**	**3220**	0.2143	3221	0.2640
	**3318**	**0.0000**	**3318**	0.0060	**3318**	**0.0000**	3320	0.3647	3321	0.7022
	**3720**	**0.0000**	**3720**	0.0269	**3720**	0.0054	**3722**	0.2124	3723	0.4086
	**3235**	**0.0000**	**3235**	**0.0000**	**3235**	**0.0000**	**3235**	0.0587	**3235**	0.1731
100 × 5	**1529**	**0.0131**	**1529**	0.2158	**1529**	0.1504	1535	0.7194	1540	1.1969
	**3646**	**0.0000**	**3646**	**0.0000**	**3646**	**0.0000**	**3646**	0.0439	**3646**	0.1015
	**4494**	**0.0000**	**4494**	**0.0000**	**4494**	**0.0000**	**4494**	0.0490	4495	0.1313
	**3826**	**0.0000**	**3826**	**0.0000**	**3826**	**0.0000**	**3826**	0.0261	**3826**	0.0497
	**1269**	**0.0709**	1270	0.1970	1271	0.5201	1272	1.8991	1289	2.6084
	**3906**	**0.0000**	**3906**	**0.0000**	**3906**	0.0410	**3906**	0.0205	**3906**	0.2125
	**1920**	**0.0417**	**1920**	0.1823	1921	0.1563	1922	0.4479	1925	0.6510
	**3744**	**0.0027**	**3744**	0.0053	**3744**	0.0374	3747	0.2404	3751	0.4006
	**3453**	**0.0000**	**3453**	0.0174	**3453**	**0.0000**	**3453**	0.0203	**3453**	0.4721
	851	**0.9220**	**846**	1.3475	856	1.9385	876	4.7518	889	7.1040
100 × 10	**4017**	**0.0149**	**4017**	0.0797	**4017**	0.0697	4037	1.0456	4081	2.3973
	**4157**	**0.0168**	**4157**	0.0433	**4157**	0.1203	4181	1.2846	4251	3.2090
	**3854**	**0.0130**	**3854**	0.1349	3855	0.2206	3866	0.4229	3875	0.9471
	**3687**	**0.0000**	**3687**	0.0434	**3687**	0.0108	**3687**	0.6618	3704	1.6273
	**3931**	**0.0000**	**3931**	0.0153	**3931**	**0.0000**	**3931**	0.3104	3942	1.0150
	**3457**	**0.0000**	**3457**	0.0579	**3457**	0.0289	**3457**	0.4484	3473	0.8360
	**3671**	**0.0054**	**3671**	0.0082	**3671**	0.1171	3683	0.5802	3689	1.0488
	**4080**	**0.0123**	**4080**	0.0368	**4080**	0.0735	**4080**	0.1642	4085	0.4240
	**3930**	**0.0000**	**3930**	**0.0000**	**3930**	**0.0000**	**3930**	0.3791	3933	0.5649
	**4318**	**0.0903**	4320	0.1065	4320	0.1667	4326	0.6901	4346	1.6350
Numbers of best *C_max_* or *RPI*	**97**	**98**	89	38	88	51	66	8	44	4
Averages of *RPI*	**0.0005**		0.0017		0.0013		0.0069		0.0132	
Variances of *RPI*	**0.000004**		0.000009		0.000011		0.000106		0.000307	

**Table 3:** The comparison test results for actual case.

HFA-VND	MBO	GATS	GAVNS	GA	LPT	LPTF
*C_max_* (min)	*C_max_* (min)	*C_max_* (min)	*C_max_* (min)	*C_max_* (min)	*C_max_* (min)	*C_max_* (min)
**1513**	1524	1536	1538	1574	1611	1618

## Data Availability

The data that support the findings of this study are available from the corresponding author, upon reasonable request.

## Abbreviations

**Parameters**:	
*m*	number of the processing stages
*n*	number of the chip types
*k*	index of processing stage, *k*∈{1,2,…,*m*}
*i*	index of chip type, *i*∈{1,2,…,*n*}
*j*	index of processing unit
*e*	index of sub-lot
***M***	set of all processing stages
***J***	set of all chip types
***M_k_***	set of all processing units at processing stage *k*
*m_k_*	number of the processing units at processing stage *k*
*l_i_*	number of sub-lots for chip type *i*
*tP_i,k_*	processing time of sub-lot for chip type *i* at processing stage *k*
*tS_i,e,k_*	starting time of sub-lot *e* for the chip type *i* at processing stage *k*
*tC_i,e,k_*	completion time of sub-lot *e* for chip type *i* at processing stage *k*
*tC_i_*	completion time for chip type *i* at processing stage *m*
*tB*	the time when the processing unit breaks down
*tR*	the time when the faulty processing unit is restored
*O_i,e,k_*	operation of sub-lot *e* for chip type *i* at processing stage *k*
***State* 1**	State set 1
***State* 2**	State set 2
***State* 3**	State set 3
*bD_i,j,k_*	1 if chip type *i* is on machine *j* at processing stage *k*, 0 otherwise
*bS_i,i’,k_*	1 if chip type *i* is superior to type *i’* on same machine at stage *k*, 0 otherwise
*Q*	a sufficiently large positive integer
**Decision variables:**	
*tS’_i,e,k_*	starting time of sub-lot *e* for chip type *i* at processing stage *k* in rescheduling
*tC’_i,e,k_*	completion time of sub-lot *e* for chip type *i* at processing stage *k* in rescheduling
*bD’_i,j,k_*	1 if chip type *i* is on machine *j* at processing stage *k* in rescheduling, 0 otherwise
*bS’_i,i’,k_*	1 if chip type *i* is superior to type *i’* on same machine at stage *k*, 0 otherwise
